# Supplementary material for: MYADM binds human parechovirus 1 and is essential for viral entry
Source: Nat Commun. 2024 Apr 24;15:3469. doi: 10.1038/s41467-024-47825-0 (PMC11043367; doi:10.1038/s41467-024-47825-0)
Supplement: Supplementary file 1 — Supplementary Information [file 41467_2024_47825_MOESM1_ESM.pdf]

MYADM binds human parechovirus 1 and is essential for viral entry

Wenjie Qiao[1], Christopher M. Richards[1], Youlim Kim[1], James R. Zengel[1], Siyuan Ding[2], Harry B. Greenberg[1,3,4], and Jan E. Carette[1*]

[1]Department of Microbiology and Immunology, Stanford University School of Medicine, Stanford, CA, USA.

[2]Department of Molecular Microbiology, Washington University School of Medicine, St. Louis, MO, USA.

[3]Division of Gastroenterology and Hepatology, Department of Medicine, Stanford University School of Medicine, Stanford, CA, USA.

[4]Department of Veterans Affairs, VA Palo Alto Health Care System, Palo Alto, CA, USA.

*Correspondence to: carette@stanford.edu

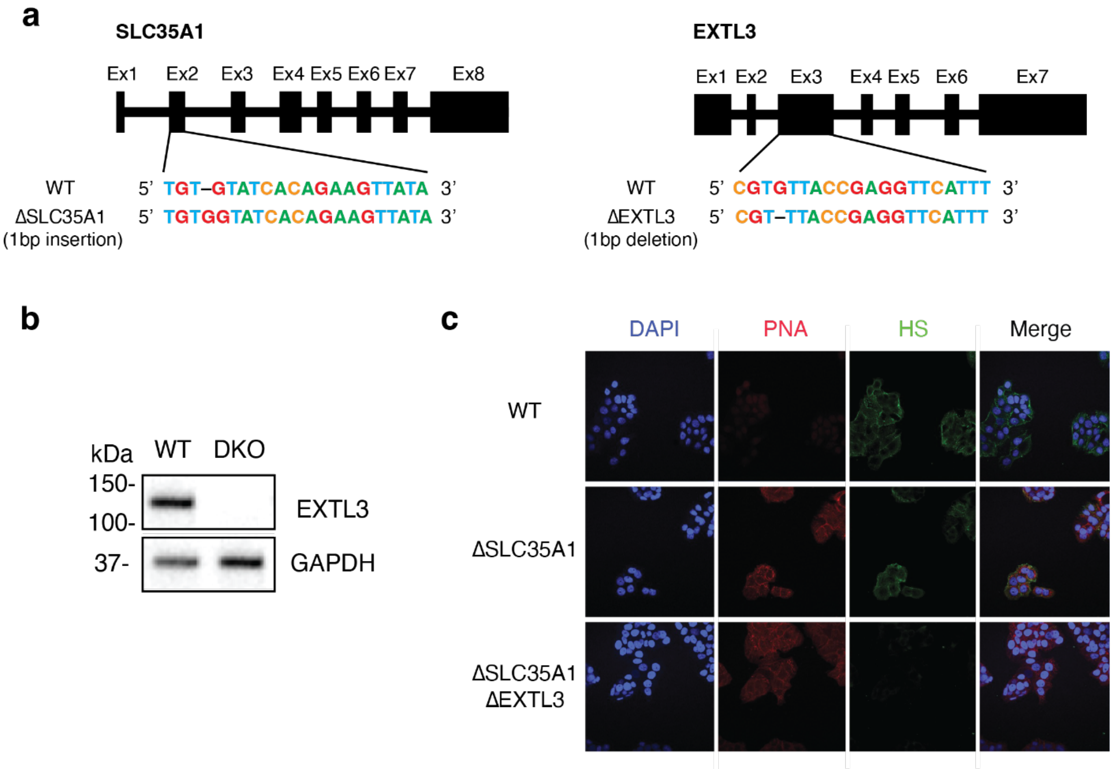

Supplementary Figure 1. HT-29 double clonal knockout cells (HT29-DKO, ΔSLC35A1 and ΔEXTL3). a, Alignment of SLC35A1 and EXTL3 isogenic knockout sequences with their respective reference sequences. **b**, Western blot analysis of EXTL3 expression in HT29-DKO cells. **c**, Immunofluorescence staining of HT-29 wild type (WT), ΔSLC35A1, and ΔSLC35A1/ΔEXTL3 cells using Rhodamine labeled Peanut agglutinin (PNA) and an antibody against heparan sulfate (HS). Loss of sialic acid biosynthesis is expected to increase PNA binding because sialic acid masks the PNA recognition site.

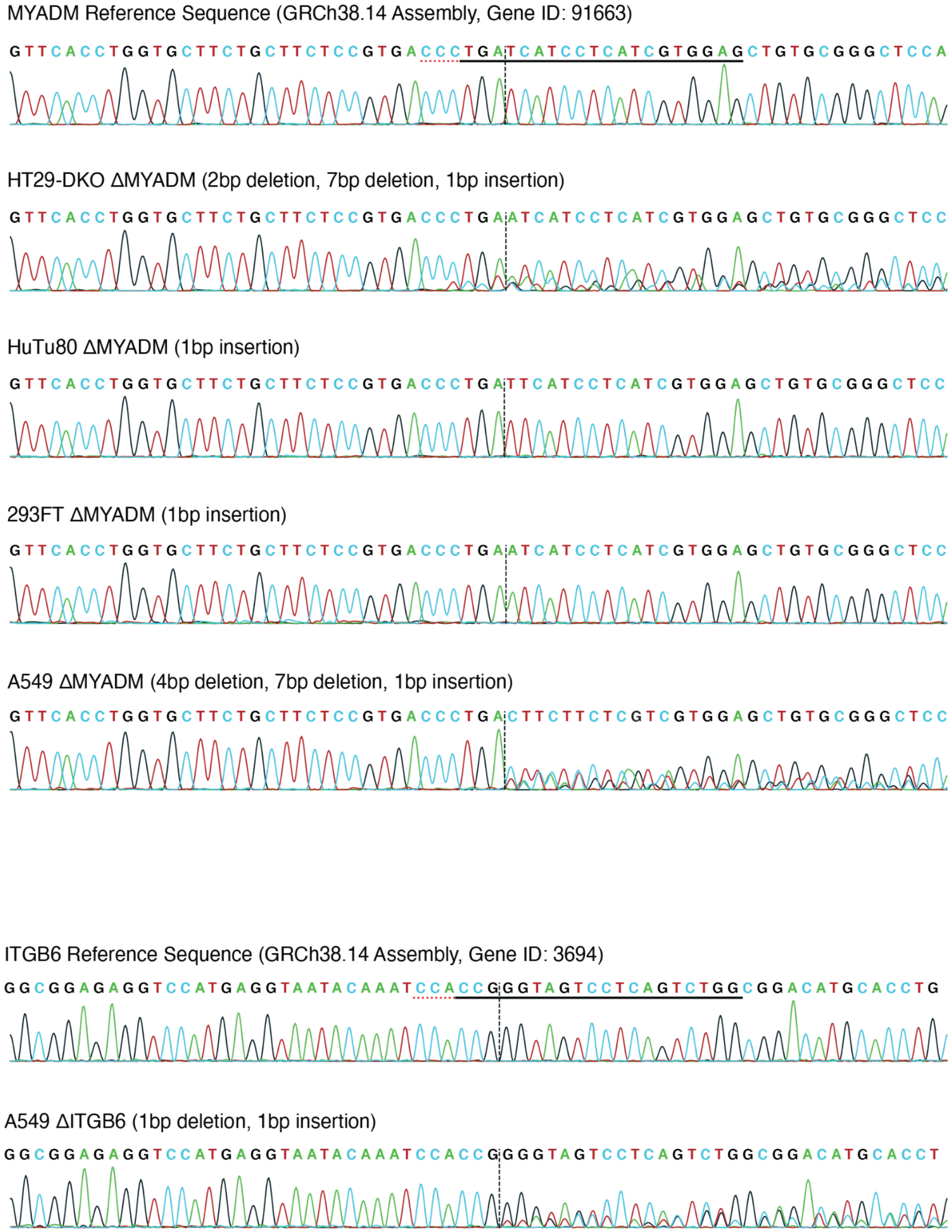

Supplementary Figure 2. Chromatograms of HT29-DKO, HuTu80, 293FT and A549 isogenic knockout cells aligned with the reference sequence. PAM sites are indicated by red underlines. The genomic DNA sequence targeted by the guide RNA (gRNA) for Cas9-mediated editing is underlined.

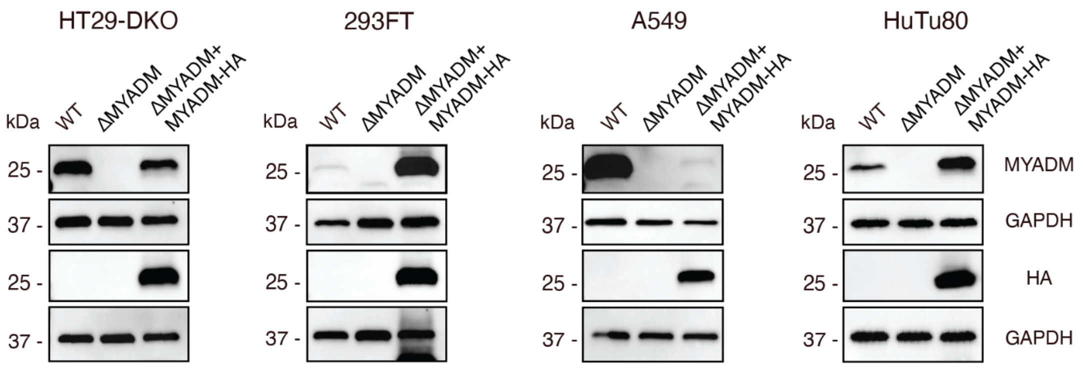

Supplementary Figure 3. Western blot analysis of MYADM expression in different cell lines. The expression level of MYADM in wild type (WT), MYADM knockout (ΔMYADM), and ΔMYADM cells expressing HA-tagged MYADM ((ΔMYADM + MYADM-HA) of multiple cell types were detected using anti-MYADM (mAb 2B12) and anti-HA antibodies. GAPDH was used as a loading control. Data from one experiment representative of two independent experiments (N = 2) are shown. Source data are provided as a Source Data file.

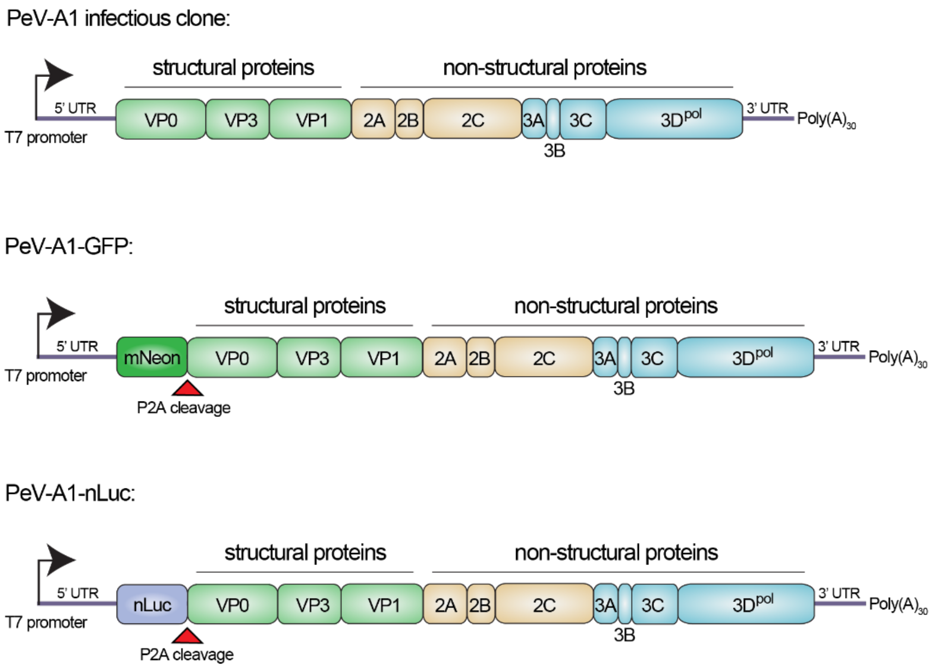

Supplementary Figure 4. Schematic of the PeV-A1 infectious clone and reporter virus constructs. The cloning strategies employed to generate the full-length infectious clone, as well as the engineered PeV-A1 reporter virus constructs expressing the mNeonGreen fluorescent protein (PeV-A1-GFP) or nanoLuciferase (PeV-A1-nLuc), are described in detail in the Materials and Methods.

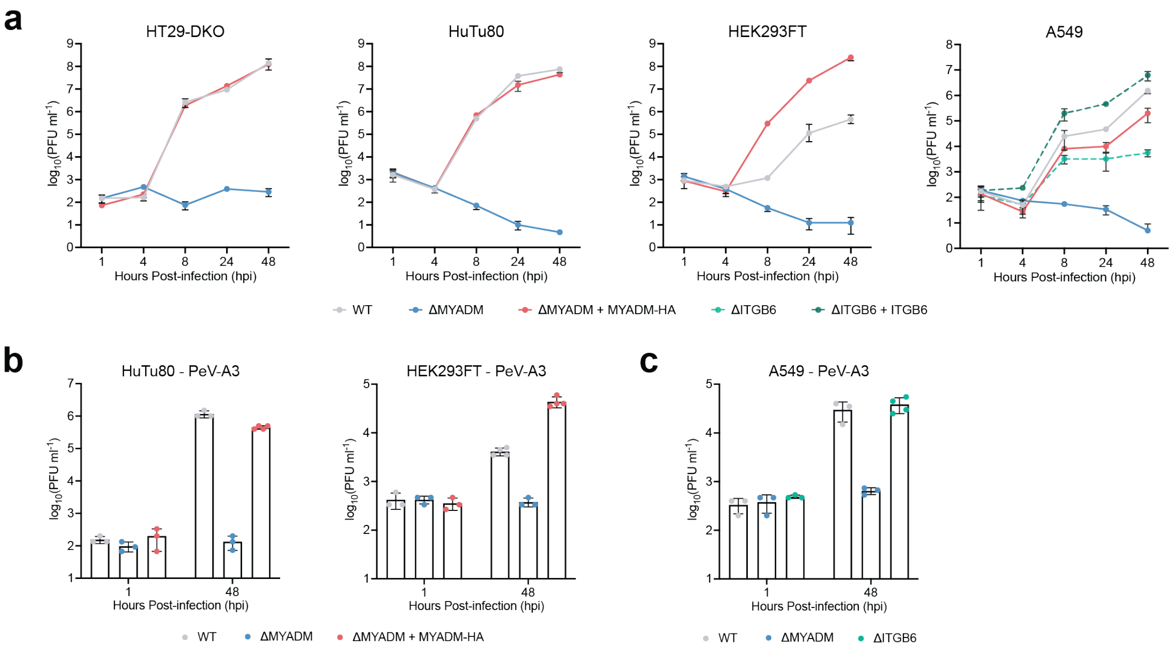

Supplementary Figure 5. Quantification of PeV-A1 and PeV-A3 infection in various MYADM or ITGB6 cell lines across multiple cell types. a, The indicated cell lines (WT, wild type; ΔMYADM, MYADM knockout; ΔMYADM + MYADM-HA, ΔMYADM cells expressing HA-tagged MYADM; ΔITGB6, ITGB6 knockout; ΔITGB6 + ITGB6, ΔITGB6 cells expressing ITGB6) of HT29-DKO, HuTu80, HEK293FT, and A549 were infected with PeV-A1 at MOI 0.1, virus titers were quantified by plaque assay at the indicated time points. Data are presented as mean values +/- SD, n = 4 biologically independent samples. **b and c**, the indicated cell lines were infected with PeV-A3 at MOI 0.1, virus titers were quantified by plaque assay at the indicated time points. Data are presented as mean values +/- SEM, n = 3 or 4 biologically independent samples. Source data are provided as a Source Data file.

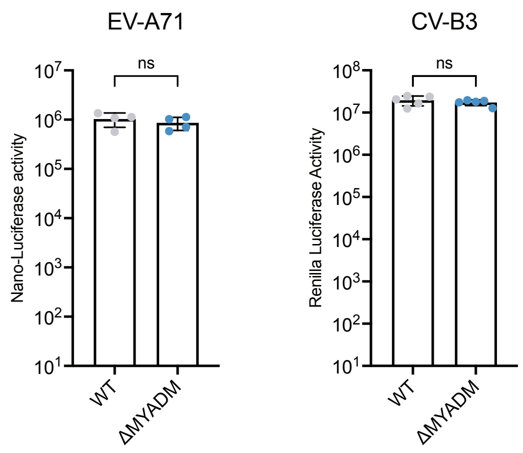

Supplementary Figure 6. MYADM dependency of different picornaviruses. Wild type (WT) and MYADM knockout (ΔMYADM) HEK293FT cells were infected with EV-A71 and CV-B3 reporter viruses at MOI 0.1 for 24 hours. Data are presented as mean values +/- SD, n = 4 or 5 biologically independent samples, unpaired two-sided t test; ns, not significant. Source data are provided as a Source Data file.

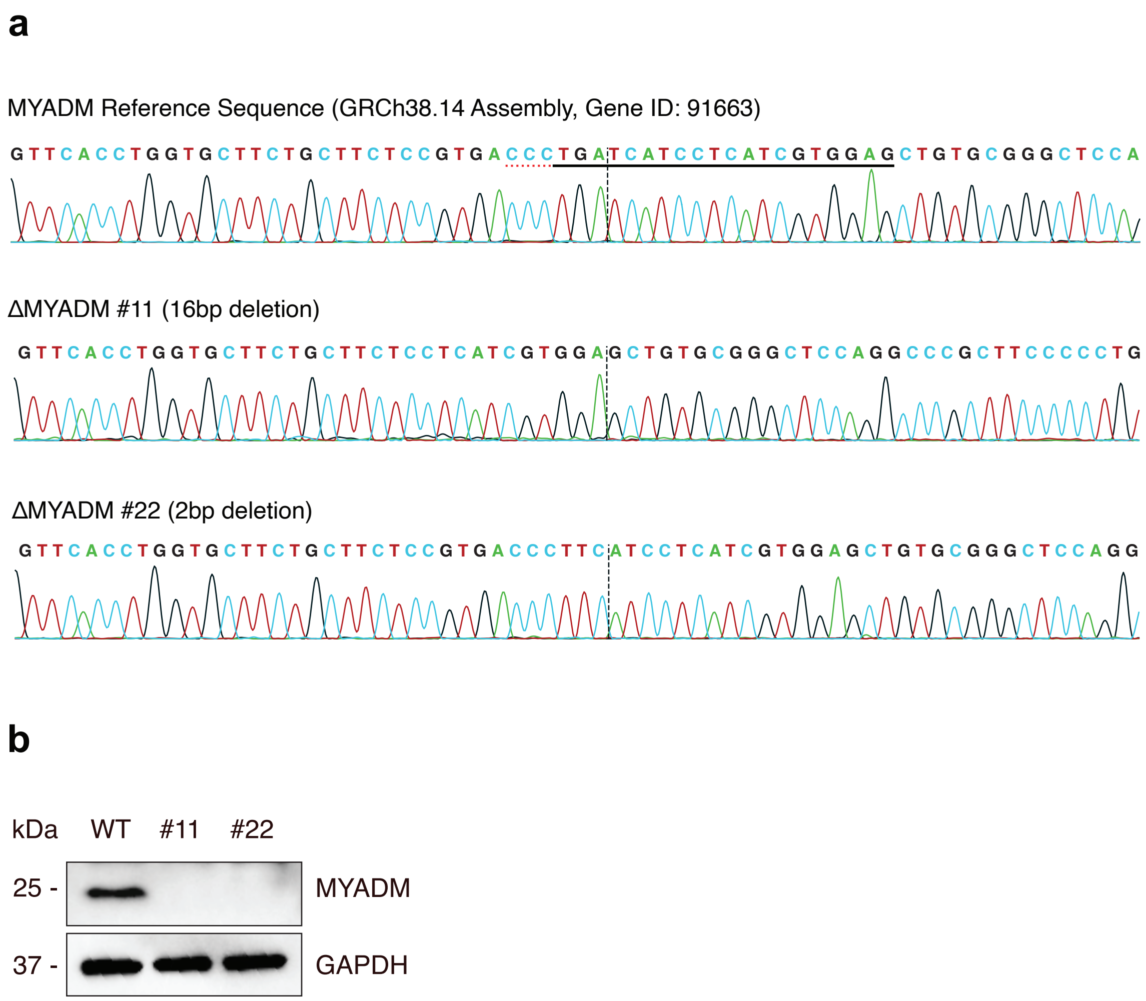

Supplementary Figure 7. Validation of human colonoids MYADM isogenic knockout. a, Human colonoids MYADM isogenic knockout chromatograms aligned with the reference sequence. PAM site is indicated by red underlines. The genomic DNA sequence targeted by the guide RNA (gRNA) for Cas9-mediated editing is underlined. **b**, Western blot analysis of MYADM expression in wild type (WT) and MYADM knockout (ΔMYADM #11, #22) organoids. Data from one experiment representative of two independent experiments (N = 2) are shown. Source data are provided as a Source Data file.

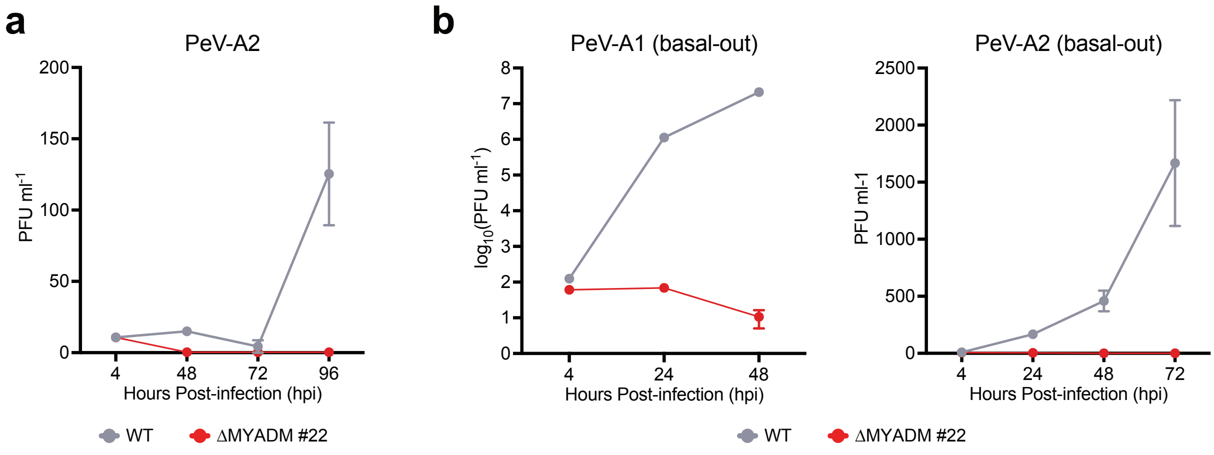

Supplementary Figure 8. The infection of PeV-A1 and PeV-A2 in primary intestinal organoids. **a**, Quantification of PeV-A2 infection in apical-out wild type (WT) and MYADM knockout (ΔMYADM #22) colonoids using plaque assay over time. Data are presented as mean values +/- SEM, n = 3 biologically independent samples. **b**, Quantification of PeV-A1 and PeV-A2 infection in basal-out WT and ΔMYADM #22 colonoids using RT-qPCR and plaque assay over time. Data are presented as mean values +/- SEM, n = 3 biologically independent samples. Source data are provided as a Source Data file.

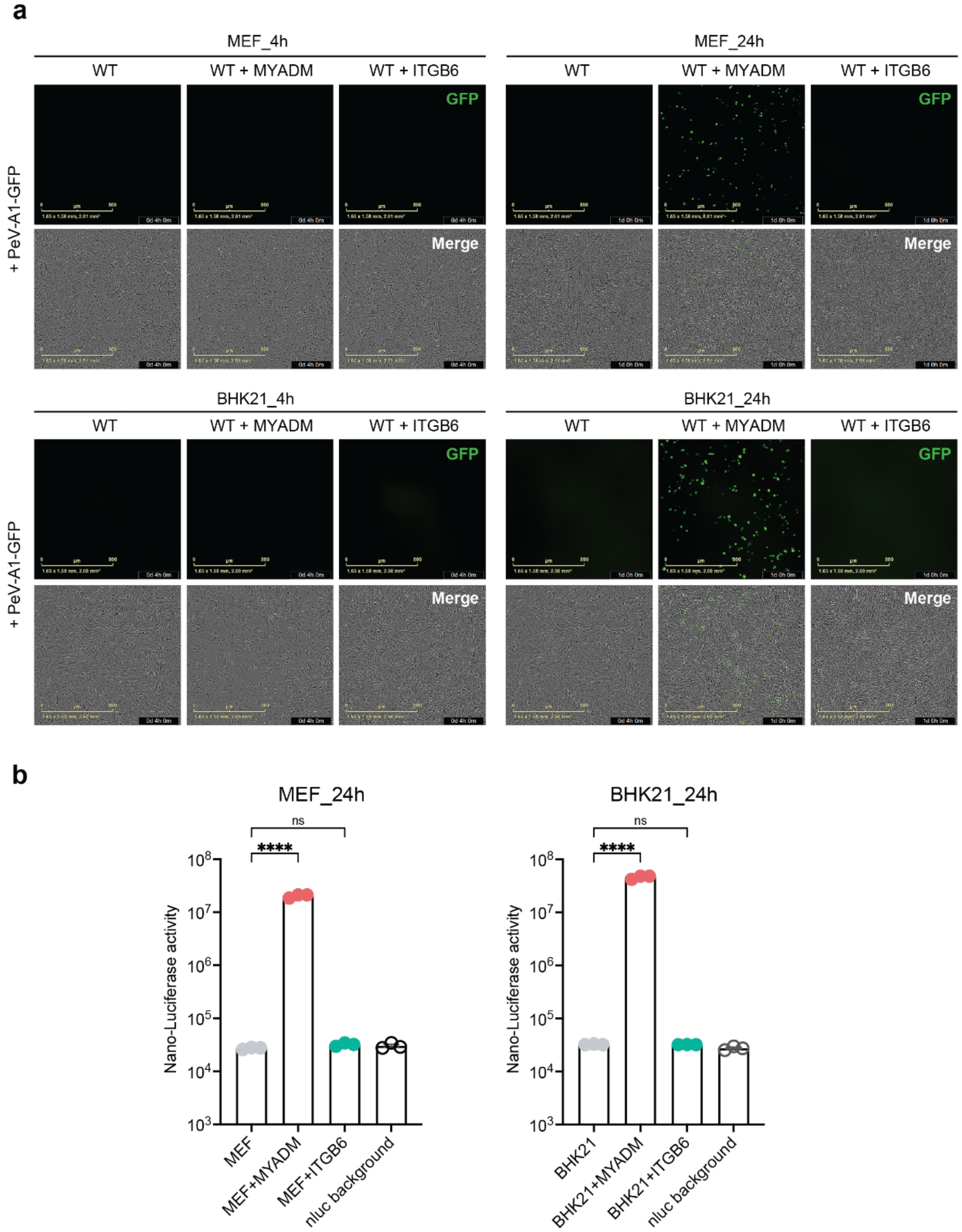

Supplementary Figure 9. MEF and BHK21 cells expressing human_MYADM are susceptible to PeV-A1 infection. a, Infection of wild type MEF/BHK21 cells (WT), human MYADM-expressing MEF/BHK21 cells (WT + MYADM), and human ITGB6-expressing

MEF/BHK21 cells (WT + ITGB6) with GFP-expressing PeV-A1 (PeV-A1-GFP, MOI 1) at the indicated time points. Representative images are shown. Quantification of the images over the full time course is shown in main data Fig. 3a. **b**, Infection of the same cell lines with nLuc-expressing PeV-A1 (PeV-A1-nLuc, MOI 1) at 24 h. PeV-A1-nLuc without cells was used as the nLuc background control. Data are presented as mean values +/- SEM, n = 3 biologically independent samples, one-way ANOVA with Šídák's multiple comparison test, ****P < 0.0001; ns, not significant. Source data are provided as a Source Data file.

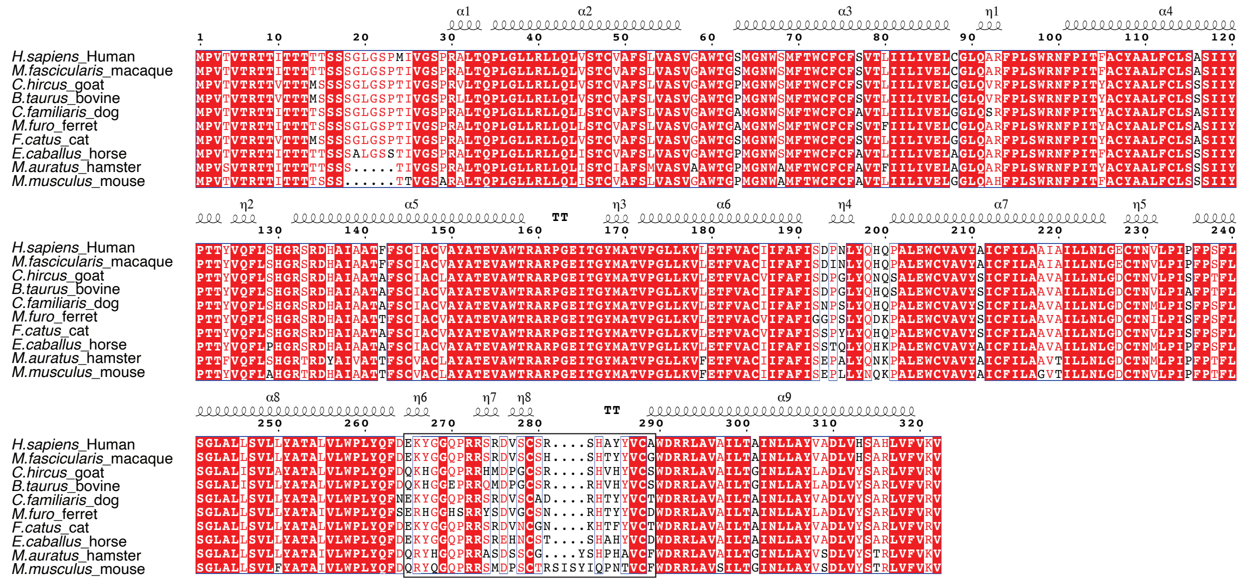

Supplementary Figure 10. Sequence alignment of full-length MYADM proteins from various mammalian species. The key extracellular loop is highlighted by a solid box and the predicted helices are shown above the sequences.

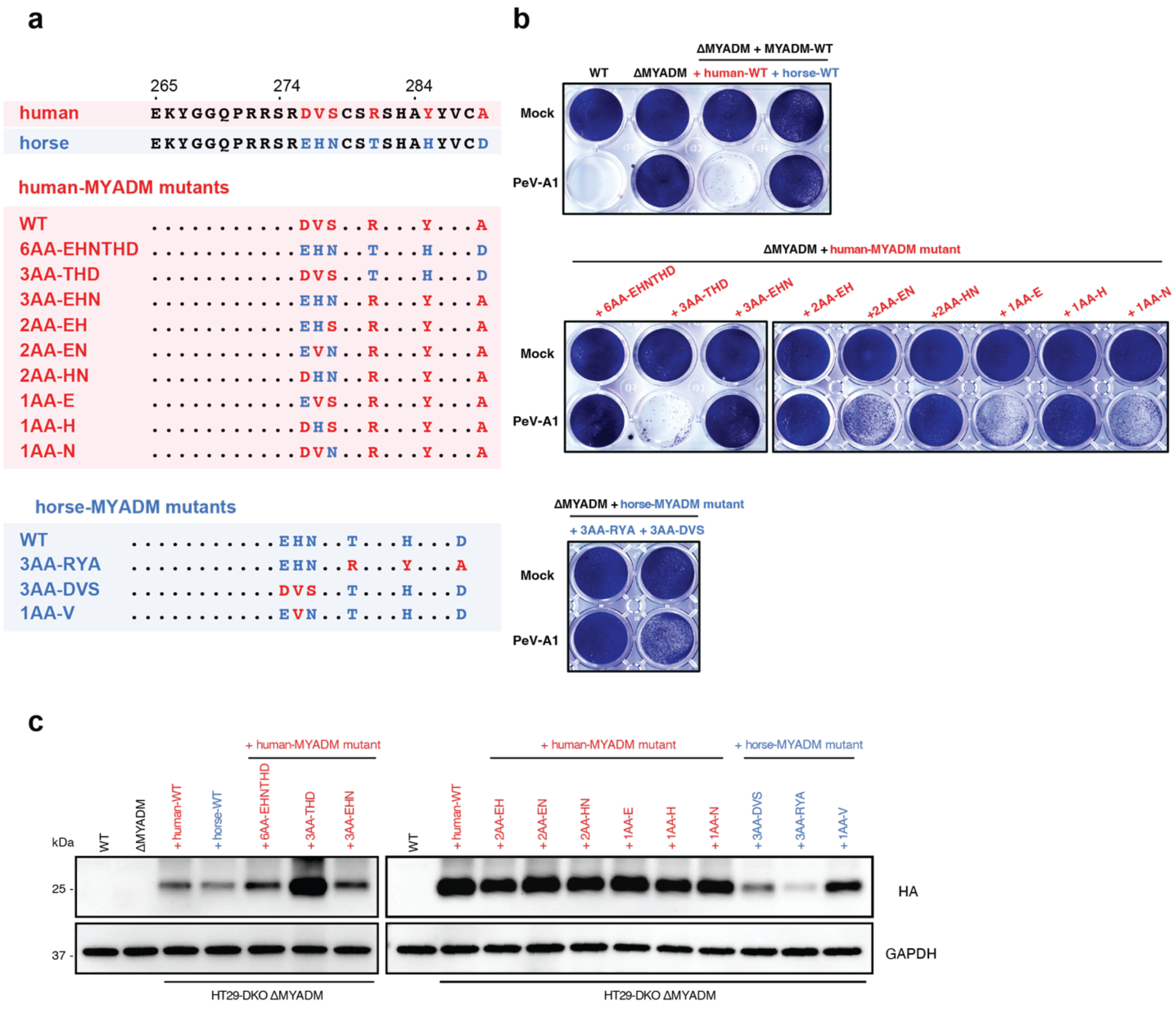

Supplementary Figure 11. Susceptibility of human MYADM and horse MYADM variants to PeV-A1 infection. a, Alignment of amino acids 265-289 in the fourth extracellular loop of human MYADM and horse MYADM proteins. The distinct amino acids are highlighted in red for human MYADM and in blue for horse MYADM. Mutations were introduced in human MYADM to replace the specific amino acids with those from horse MYADM, and vice versa, as indicated. **b**, Crystal violet staining of HT29-DKO cell lines expressing human MYADM or horse MYADM variants, infected with PeV-A1 at MOI 0.5. **c**, Western blot analysis demonstrating the expression of the corresponding HA-tagged MYADM variants in HT29-DKO ΔMYADM cells. GAPDH was

used as a loading control. Data from one experiment representative of two independent experiments (N = 2) are shown. Source data are provided as a Source Data file.

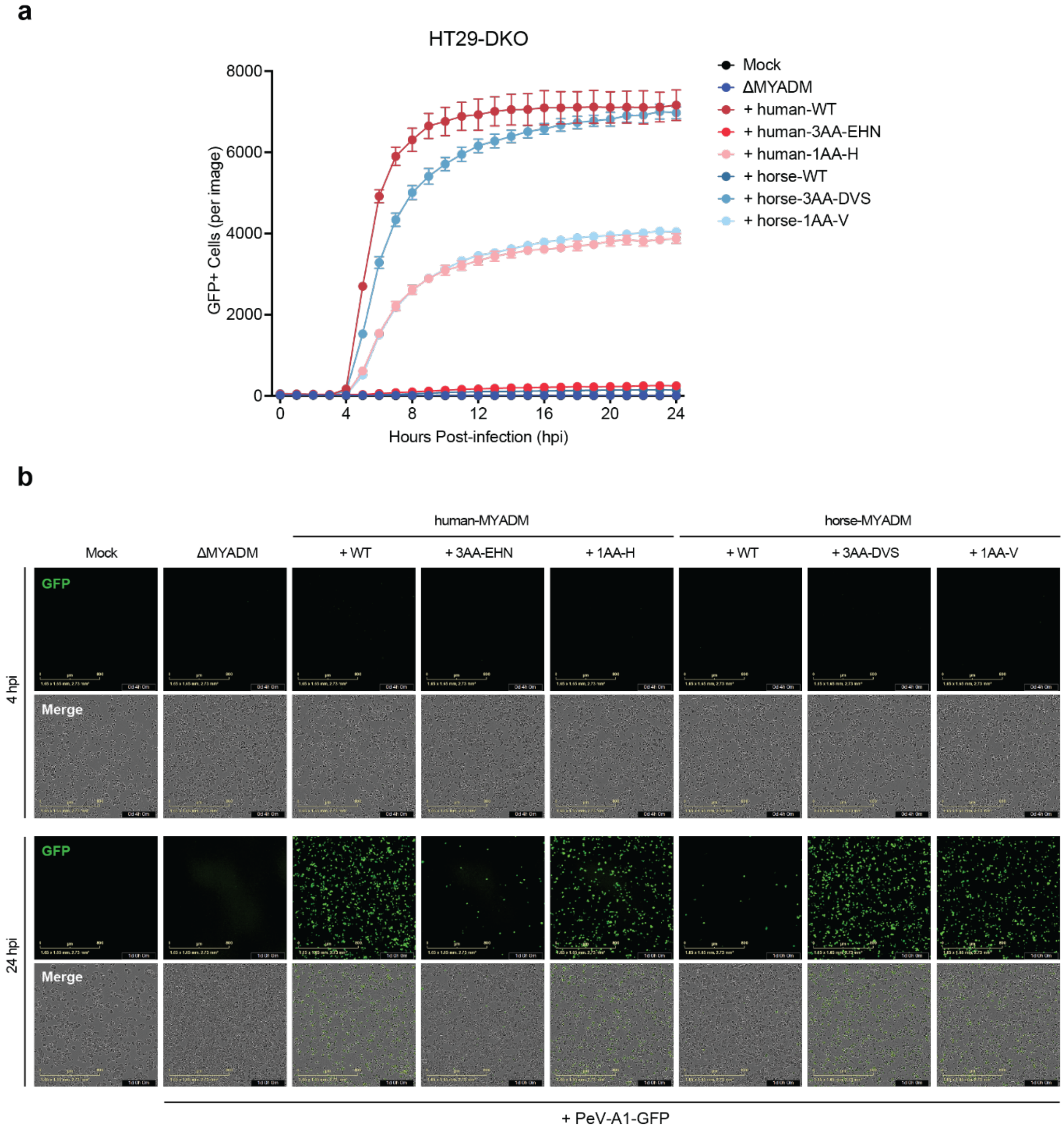

Supplementary Figure 12. Susceptibility of human and horse MYADM and their indicated mutants to PeV-A1 infection tested in HT29-DKO MYADM knockout cells. a, Time course of GFP-expressing PeV-A1 (PeV-A1-GFP, MOI 0.1) infection at 24 hours post-infection (hpi), quantified by the number of GFP positive cells per image. Uninfected cells were used as Mock control. Data are presented as mean values +/- SEM, n = 3 biologically independent samples. **b**,

Representative images of PeV-A1-GFP infection at 4 hpi or 24 hpi at MOI 0.1. Source data are provided as a Source Data file.

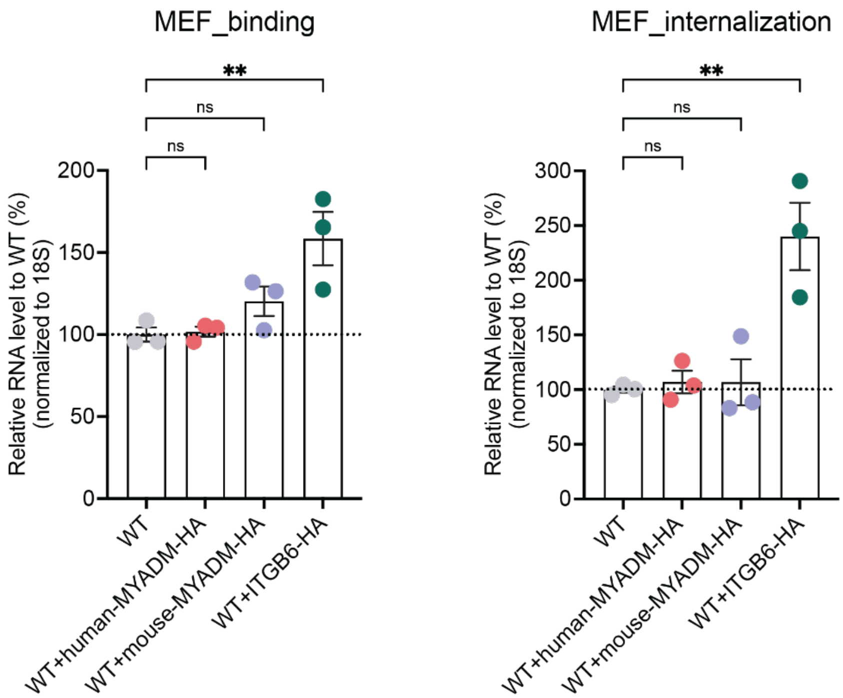

Supplementary Figure 13. PeV-A1 binding and internalization assays conducted with MEF cells. MEF wild type (WT) cells, MEF cells expressing HA-tagged human MYADM, mouse MYADM, and human ITGB6 were incubated with PeV-A1 at 4 °C for 1 h (binding assay), or followed by incubation at 37°C for 1 h after removal of unbound virus (internalization assay). Viral RNA levels were measured by RT-qPCR and normalized to 18S. Data are presented as mean values +/- SEM, n= 3 biologically independent samples, one-way ANOVA with Šídák's multiple comparison test, **$p < 0.01$; ns, not significant. Source data are provided as a Source Data file.

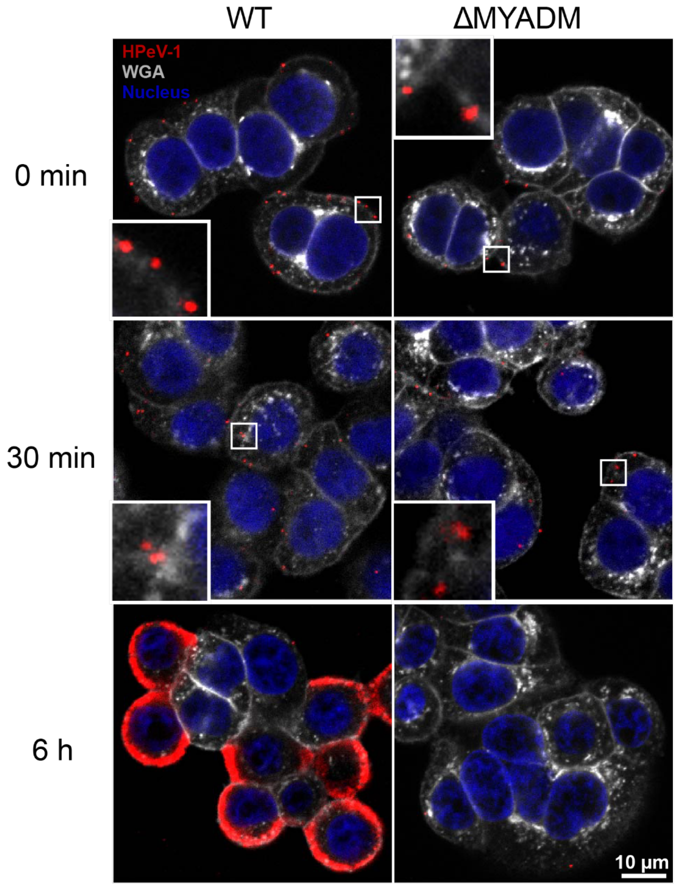

Supplementary Figure 14. Fluorescence *in situ* hybridization of PeV-A1 genomic RNA (red) in wild type (WT) or ΔMYADM HT29-DKO cells visualized using confocal microscopy. Cells were incubated with PeV-A1 (MOI of ~100) for 1 h on ice, followed by incubation at 37 °C for 0 min, 30 min, or 6 h. Glycoproteins and glycolipids present in cell membranes and in the secretory pathway were labeled with wheat germ agglutinin lectin (WGA, white), and DNA was stained using Hoechst 33342 (blue). Insets represent magnifications of the square boxes. Scale bar, 10 µm. Signal at 0 and 30 min represents incoming viral RNA while the increased signal at 6 h in WT cells is due to genome amplification.

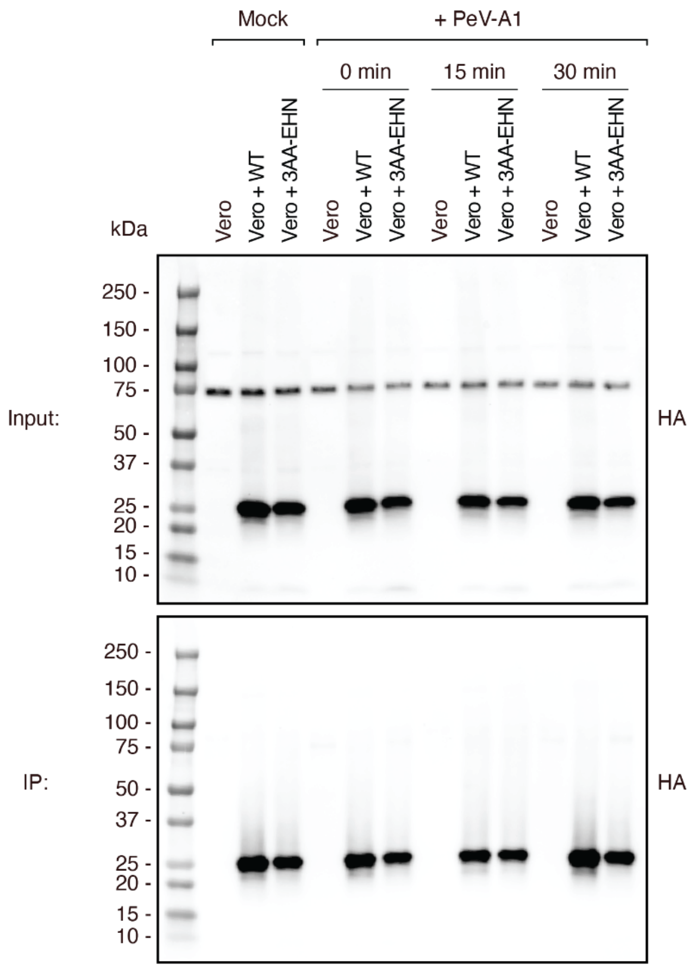

Supplementary Figure 15. Western blot analysis of HA-tagged MYADM variants after immunoprecipitation in the time point virus particle binding assay. Vero cells, Vero cells expressing HA-tagged human MYADM-WT (Vero + WT), and MYADM-3AA-EHN mutant (Vero + 3AA-EHN) were incubated with concentrated PeV-A1 at 4 °C for 1 h, followed by incubation at 37 °C for 0, 15, or 30 min after removing unbound viruses. Cells without virus infection were included as the Mock control. Cells were lysed and immunoprecipitated with anti-HA beads. The protein expression of MYADM in the total lysate (Input) and after immunoprecipitation (IP) was analyzed by western blot using an anti-HA antibody. Data from one experiment representative of three independent experiments (N = 3) are shown.

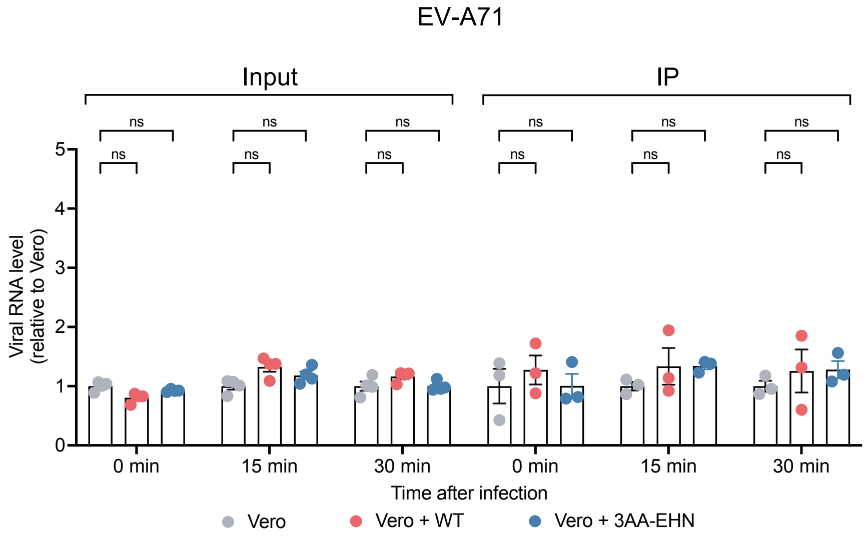

Supplementary Figure 16. Time point virus particle binding assay of EV-A71. Vero cells, Vero cells expressing HA-tagged human MYADM (Vero + WT), and 3AA-EHN mutant (Vero + 3AA-EHN) were incubated with concentrated EV-A71 at 4 °C for 1 h, followed by incubation at 37 °C for 0, 15, or 30 min after removing unbound viruses. Cells were lysed (Input) and immunoprecipitated with anti-HA beads (IP). Viral RNA levels were measured by RT-qPCR. Data are presented as mean values +/- SEM, n = 3 or 4 biologically independent samples, two-way ANOVA with Šídák’s multiple comparison test; ns, not significant). Source data are provided as a Source Data file.

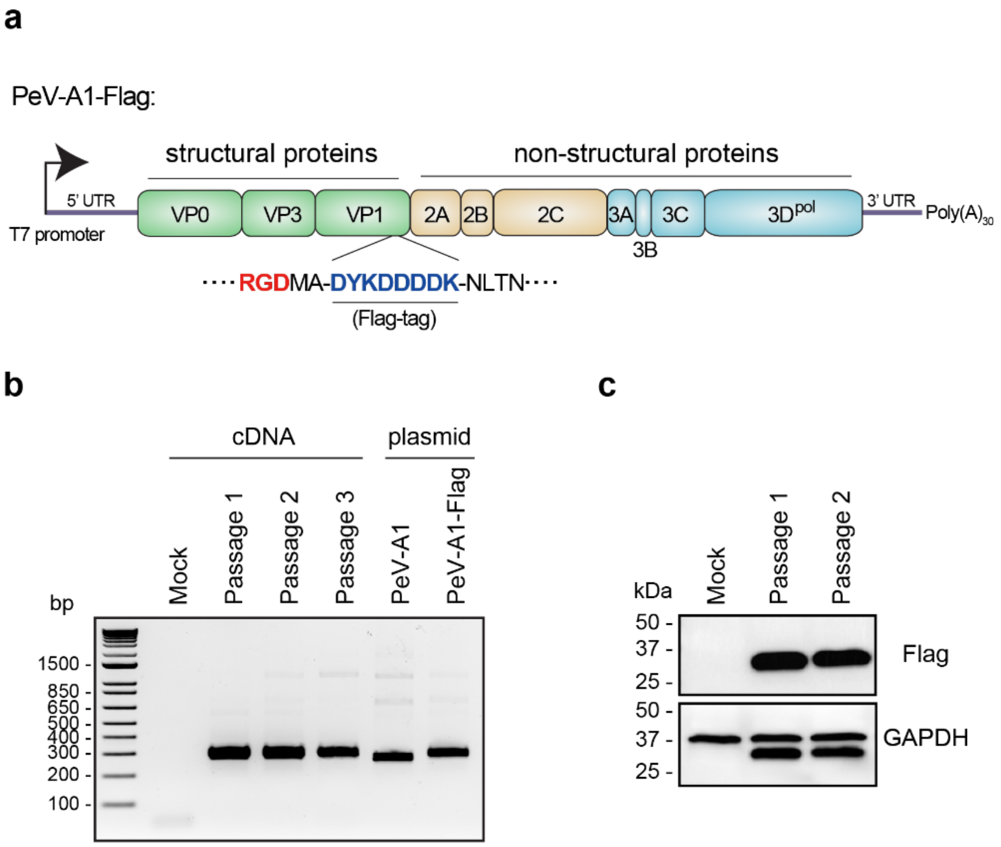

Supplementary Figure 17. Generation of Flag-tagged PeV-A1 virus. a, Schematic of the PeV-A1-Flag construct, in which the Flag sequence was inserted into the structural protein VP1. The cloning strategy is described in detail in the Materials and Methods. **b**, The stability of the Flag-tag insertion was validated by passaging PeV-A1-Flag on Vero cells, and presence of the Flag sequence was confirmed by RT-PCR. The PCR products were compared to the fragment amplified from the PeV-A1 and PeV-A1-Flag plasmid constructs on a DNA gel. **c**, Detection of the Flag-tagged VP1 protein in virus infected Vero cells (passage 1 and 2) by Western blot. Data from one experiment representative of two independent experiments (N = 2) are shown. Source data are provided as a Source Data file.

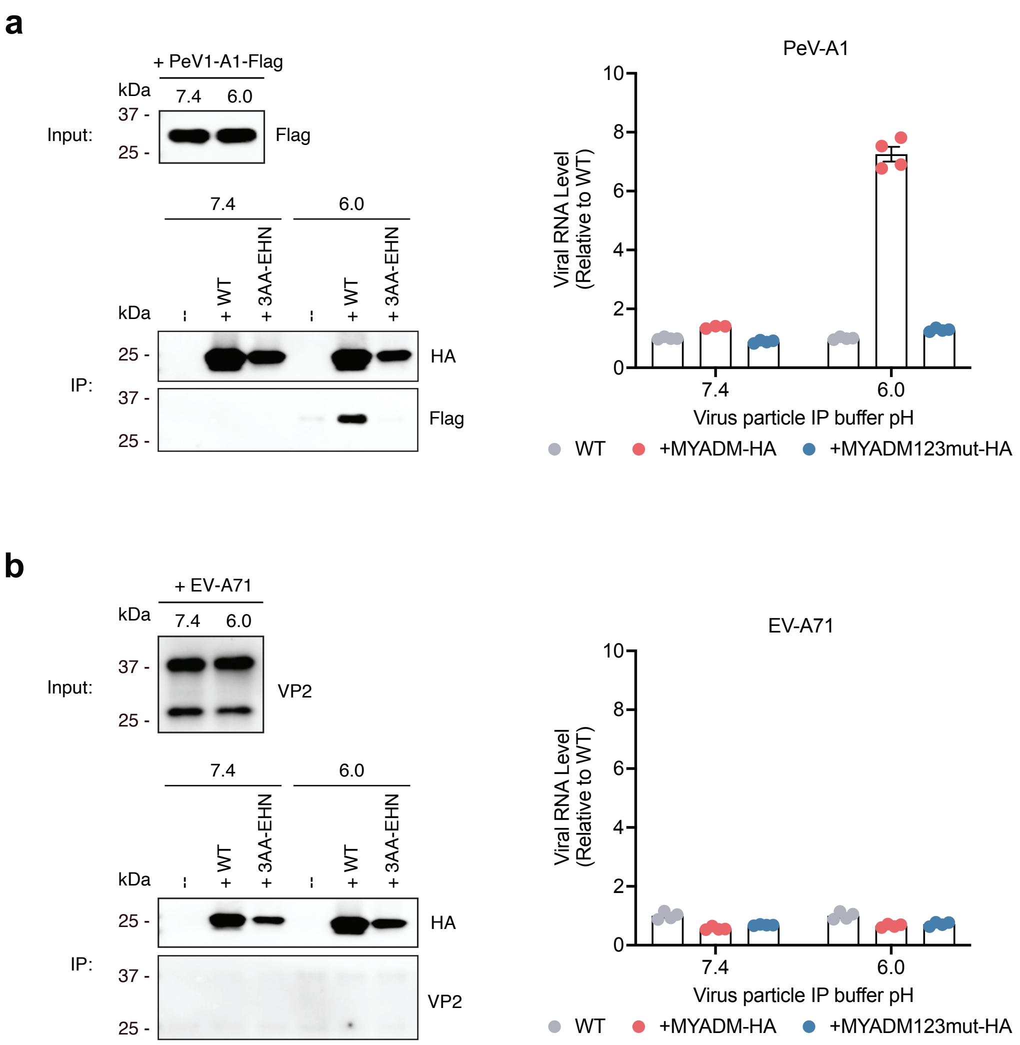

Supplementary Figure 18. The pH-dependent virus particle binding assay of MYADM with PeV-A1-Flag (a) and EV-A71 (b) viruses. Left, western blot analysis of the input viruses in NETN buffers with pH 7.4 and 6.0, as well as the final immunoprecipitates (IP) of HA-tagged MYADM proteins and viral proteins. Right, quantification of viral RNA levels after immunoprecipitation using RT-qPCR. Data are presented as mean values +/- SEM, n = 3 or 4 biologically independent samples. Data from one experiment representative of two independent experiments (N = 2) are shown. Source data are provided as a Source Data file.
