# Supplementary material for: MYADM binds human parechovirus 1 and is essential for viral entry
Source: Nat Commun. 2024 Apr 24;15:3469. doi: 10.1038/s41467-024-47825-0 (PMC11043367; doi:10.1038/s41467-024-47825-0)
Supplement: Supplementary file 3 — Description of Additional Supplementary Files [file 41467_2024_47825_MOESM3_ESM.docx]

**MYADM binds human parechovirus 1 and is essential for viral entry**

Wenjie Qiao^1^, Christopher M. Richards^1^, Youlim Kim^1^, James R. Zengel^1^, Siyuan Ding^2^, Harry B. Greenberg^1,3,4^, and Jan E. Carette^1*^

^1^Department of Microbiology and Immunology, Stanford University School of Medicine, Stanford, CA, USA.

^2^Department of Molecular Microbiology, Washington University School of Medicine, St. Louis, MO, USA.

^3^Division of Gastroenterology and Hepatology, Department of Medicine, Stanford University School of Medicine, Stanford, CA, USA.

^4^Department of Veterans Affairs, VA Palo Alto Health Care System, Palo Alto, CA, USA.

*Correspondence to: carette@stanford.edu

**Supplementary Data 1**

**Dataset of HT29-DKO PeV-A1 and PeV-A2 CRISPR screens.** Genome-scale CRISPR screen datasets were analyzed using MAGeCK algorithm (one-sided P-value).
