# Supplementary material for: MYADM binds human parechovirus 1 and is essential for viral entry
Source: Nat Commun. 2024 Apr 24;15:3469. doi: 10.1038/s41467-024-47825-0 (PMC11043367; doi:10.1038/s41467-024-47825-0)
Supplement: Supplementary file 5 — Reporting Summary [file 41467_2024_47825_MOESM5_ESM.pdf]

nature portfolio

Corresponding author(s): Jan Carette

Last updated by author(s): Mar 28, 2024

Reporting Summary

Nature Portfolio wishes to improve the reproducibility of the work that we publish. This form provides structure for consistency and transparency in reporting. For further information on Nature Portfolio policies, see our Editorial Policies and the Editorial Policy Checklist.

Statistics

For all statistical analyses, confirm that the following items are present in the figure legend, table legend, main text, or Methods section.

n/a	Confirmed	
☐	☒	The exact sample size (*n*) for each experimental group/condition, given as a discrete number and unit of measurement
☐	☒	A statement on whether measurements were taken from distinct samples or whether the same sample was measured repeatedly
☐	☒	The statistical test(s) used AND whether they are one- or two-sided<br>*Only common tests should be described solely by name; describe more complex techniques in the Methods section.*
☒	☐	A description of all covariates tested
☒	☐	A description of any assumptions or corrections, such as tests of normality and adjustment for multiple comparisons
☐	☒	A full description of the statistical parameters including central tendency (e.g. means) or other basic estimates (e.g. regression coefficient) AND variation (e.g. standard deviation) or associated estimates of uncertainty (e.g. confidence intervals)
☐	☒	For null hypothesis testing, the test statistic (e.g. *F*, *t*, *r*) with confidence intervals, effect sizes, degrees of freedom and *P* value noted<br>*Give P values as exact values whenever suitable.*
☒	☐	For Bayesian analysis, information on the choice of priors and Markov chain Monte Carlo settings
☒	☐	For hierarchical and complex designs, identification of the appropriate level for tests and full reporting of outcomes
☒	☐	Estimates of effect sizes (e.g. Cohen's *d*, Pearson's *r*), indicating how they were calculated

Our web collection on statistics for biologists contains articles on many of the points above.

Software and code

Policy information about availability of computer code

Data collection	After genomic DNA extraction, sgRNAs were enriched, amplified, and sequenced using a HiSeq instrument lane (Illumina) via Novogene.<br>Images were acquired with an inverted confocal microscope (Zeiss LSM 800) and processed with ZEN software (Zeiss).
Data analysis	sgRNA sequences were analyzed using a published computational tool (MAGeCK v0.5.9.2).<br>Immunofluorescence images were processed using the FIJI software.<br>Data representation and statistical analysis were performed using GraphPad Prism 10 software.<br>Protein alignment was performed using https://www.genome.jp/tools-bin/clustalw. Cladogram was generated using http://phylogeny.lirmm.fr/phylo_cgi/index.cgi.

For manuscripts utilizing custom algorithms or software that are central to the research but not yet described in published literature, software must be made available to editors and reviewers. We strongly encourage code deposition in a community repository (e.g. GitHub). See the Nature Portfolio guidelines for submitting code & software for further information.

Data

Policy information about availability of data

All manuscripts must include a data availability statement. This statement should provide the following information, where applicable:

- Accession codes, unique identifiers, or web links for publicly available datasets
- A description of any restrictions on data availability
- For clinical datasets or third party data, please ensure that the statement adheres to our policy

The FASTQ files for CRISPR screens generated in this study have been deposited in ArrayExpress under accession code E-MTAB-13894. The Mageck analysis of the CRISPR screens are provided as Supplementary Data 1. MYADM protein sequences: Homo sapiens (NP_001018654.1), Mus musculus (NP_001087233.1), Equus caballus (XP_023506108.1), Canis lupus familiaris (XP_003638852.1), Felis catus (XP_003997497.1), Mesocricetus auratus (XP_005084208.1), Macaca fascicularis (XP_005590325.1), Capra hircus (XP_017918523.1), Bos taurus (XP_027370620.1), and Mustela putorius furo (XP_012905045.1), are available from NCBI database. Datasets analysed during the current study are appended as supplementary data. Source data are provided with this paper.

Research involving human participants, their data, or biological material

Policy information about studies with human participants or human data. See also policy information about sex, gender (identity/presentation), and sexual orientation and race, ethnicity and racism.

Reporting on sex and gender	Colon organoids were derived from de-identified, surgically obtained human gastrointestinal tissue. Age and sex of the patients were not collected.
Reporting on race, ethnicity, or other socially relevant groupings	Colon organoids were derived from de-identified, surgically obtained human gastrointestinal tissue.
Population characteristics	Colon organoids were derived from de-identified, surgically obtained human gastrointestinal tissue. Age and sex of the patients were not collected.
Recruitment	Samples were taken without a particular targeted or planned enrollment.
Ethics oversight	Tissues were obtained through the Stanford Tissue Bank with patient consent and approval from the Stanford University Institutional Review Board.

Note that full information on the approval of the study protocol must also be provided in the manuscript.

Field-specific reporting

Please select the one below that is the best fit for your research. If you are not sure, read the appropriate sections before making your selection.

☒ Life sciences ☐ Behavioural & social sciences ☐ Ecological, evolutionary & environmental sciences

For a reference copy of the document with all sections, see nature.com/documents/nr-reporting-summary-flat.pdf

Life sciences study design

All studies must disclose on these points even when the disclosure is negative.

Sample size	All experiments with statistical analysis were performed with at least three biological replicates.
Data exclusions	No data was excluded.
Replication	All experimental data were reliably produced. All experiments had at least 2 independent experiments and 3 biologically independent samples, which is specified in the figure legends.
Randomization	Not relevant to this study, samples were not allocated into experimental groups.
Blinding	Not relevant to this study, no group allocations in this study.

Reporting for specific materials, systems and methods

We require information from authors about some types of materials, experimental systems and methods used in many studies. Here, indicate whether each material, system or method listed is relevant to your study. If you are not sure if a list item applies to your research, read the appropriate section before selecting a response.

Materials & experimental systems

n/a	Involved in the study
☐	☒ Antibodies
☐	☒ Eukaryotic cell lines
☒	☐ Palaeontology and archaeology
☒	☐ Animals and other organisms
☒	☐ Clinical data
☒	☐ Dual use research of concern
☒	☐ Plants

Methods

n/a	Involved in the study
☒	☐ ChIP-seq
☒	☐ Flow cytometry
☒	☐ MRI-based neuroimaging

Antibodies

Antibodies used

Anti-dsRNA Antibody, clone rJ2 (1:100, Sigma-Aldrich, MABE1134); Alexa Fluor 488 Goat anti-Mouse secondary antibody (1:500, ThermoFisher, A-11001); Alexa Fluor 594 phalloidin (ThermoFisher, A12381); Alexa Fluor 488 anti-GFP polyclonal antibody (1:100, ThermoFisher, A-21311); anti-EXTL3 antibody (1:1000, Santa Cruz Biotechnology, clone G-5, sc-271986); anti-heparan sulfate (1:100, Amsbio, clone F58-10E4, 370255-S); MYADM antibody (1:500, mAb 2B12, gift from Miguel A. Alonso); goat anti-mouse IgG antibody (1:5000, GeneTex, GTX213111-01); HRP-conjugated GAPDH antibody (1:5000, GeneTex, GTX627408-01); HA Tag Polyclonal Antibody (1:1000, ThermoFisher, 14-6756-81); goat anti-rabbit IgG antibody (1:5000, GeneTex, GTX213110-01); Flag Tag Antibody (1:1000, Cell Signaling Technology, 2368S)

Validation

mouse Anti-dsRNA Antibody, clone rJ2 (Sigma-Aldrich, MABE1134);commercially validated by IF and ICC, https://www.sigmaaldrich.com/US/en/product/mm/mabe1134
Alexa Fluor 594 phalloidin (ThermoFisher, A12381); commercially validated by IF, https://www.thermofisher.com/order/catalog/product/A12381
Alexa Fluor 488 anti-GFP polyclonal antibody (ThermoFisher, A-21311); commercially validated by WB, IHC, ICC, IF, Flow, ChIP and Misc, https://www.thermofisher.com/antibody/product/GFP-Antibody-Polyclonal/A-21311
mouse anti-EXTL3 antibody (Santa Cruz Biotechnology, clone G-5, sc-271986); commercially validated by WB, https://www.scbt.com/p/extl3-antibody-g-5?requestFrom=search
mouse anti-heparan sulfate (Amsbio, clone F58-10E4, 370255-S); validated by WB and IHC (PMID: 1385449), https://www.amsbio.com/ab-heparan-sulfate-purified-clone-f58-10e4-50ug-pack-370255-s
mouse anti-MYADM antibody (mAb 2B12); validated by WB (PMID: 21325632), gift from Miguel A. Alonso
HRP-conjugated GAPDH antibody (GTX627408-01); commercially validated by WB and IHC-P, https://www.genetex.com/Product/Detail/GAPDH-antibody-GT239-HRP/GTX627408-01
rabbit HA Tag Polyclonal Antibody (ThermoFisher, 14-6756-81); commercially validated by WB, IHC, ICC, ELISA, IP and ChIP, https://www.thermofisher.com/antibody/product/HA-Tag-Antibody-Polyclonal/14-6756-81
rabbit Flag Tag Antibody (Cell Signaling Technology, 2368S); commercially validated by WB and Flow, https://www.cellsignal.com/products/primary-antibodies/dykddddk-tag-antibody-binds-to-same-epitope-as-sigma-aldrich-anti-flag-m2-antibody/2368

Eukaryotic cell lines

Policy information about cell lines and Sex and Gender in Research

Cell line source(s)

HT-29 (ATCC, HTB-38); A549 (ATCC, CCL-185); 293FT (Thermo Fisher, #R70007); HuTu80 (ATCC, HTB-40); Vero (ATCC, CCL-81); BHK21 (ATCC, CCL-10); MEF cells (described in PMID: 36074821)

Authentication

Commercially validated cell lines were obtained from ATCC and other sources and grew and performed as expected. Morphology of each cell line was assessed by microscopy.

Mycoplasma contamination

The cell lines were not tested for mycoplasma contamination.

Commonly misidentified lines
(See ICLAC register)

This study did not involve any commonly misidentified cell lines.
